# RNA interference-based resistance against a legume mastrevirus

**DOI:** 10.1186/1743-422X-8-499

**Published:** 2011-11-02

**Authors:** Nazia Nahid, Imran Amin, Rob W Briddon, Shahid Mansoor

**Affiliations:** 1 Agricultural Biotechnology Division, National Institute for Biotechnology and Genetic Engineering (NIBGE), P O Box 577, Jhang Road, Faisalabad, Pakistan

## Abstract

**Background:**

RNA interference (RNAi) is a homology-dependant gene silencing mechanism and has been widely used to engineer resistance in plants against RNA viruses. However, its usefulness in delivering resistance against plant DNA viruses belonging to family *Geminiviridae *is still being debated. Although the RNAi approach has been shown, using a transient assay, to be useful in countering monocotyledonous plant-infecting geminiviruses of the genus *Mastrevirus*, it has yet to be investigated as a means of delivering resistance to dicot-infecting mastreviruses. *Chickpea chlorotic dwarf Pakistan virus *(CpCDPKV) is a legume-infecting mastrevirus that affects chickpea and other leguminous crops in Pakistan.

**Results:**

Here a hairpin (hp)RNAi construct containing sequences encompassing part of replication-associated protein gene, intergenic region and part of the movement protein gene of CpCDPKV under the control of the *Cauliflower mosaic virus *35S promoter has been produced and stably transformed into *Nicotiana benthamiana*. Plants harboring the hairpin construct were challenged with CpCDPKV. All non-transgenic *N. benthamiana *plants developed symptoms of CpCDPKV infection within two weeks post-inoculation. In contrast, none of the inoculated transgenic plants showed symptoms of infection and no viral DNA could be detected by Southern hybridization. A real-time quantitative PCR analysis identified very low-level accumulation of viral DNA in the inoculated transgenic plants.

**Conclusions:**

The results presented show that the RNAi-based resistance strategy is useful in protecting plants from a dicot-infecting mastrevirus. The very low levels of virus detected in plant tissue of transgenic plants distal to the inoculation site suggest that virus movement and/or viral replication was impaired leading to plants that showed no discernible signs of virus infection.

## Introduction

RNA interference (RNAi) is a homology-dependent mechanism that involves the specific degradation of cellular RNA by a complex of enzymes. The phenomenon was first discovered in plants and was called "post-transcriptional gene silencing" (PTGS) [1]. RNAi is involved in controlling developmental processes and also as a defense against viruses, transposons and foreign nucleic acids. The key role in RNAi is played by small RNAs [known as short interfering RNA (siRNA) and micro RNA (miRNA)], which act as effectors of silencing [2]. Plants recognize dsRNA as foreign/aberrant and this is cleaved into 21-26 nt siRNAs by a ribonuclease III-like enzyme called Dicer [3]. One strand of the siRNA is incorporated into a ribonuclease complex known as the RNA-induced silencing complex (RISC) and serves as the guide for sequence-specific degradation of homologous mRNAs [4]. siRNAs homologous to promoter regions of target genes induce transcriptional gene silencing (TGS) which results in promoter methylation and consequent inhibition of transcription. siRNAs homologous to coding regions induce PTGS, which results in sequence-specific RNA degradation. PTGS and TGS are mechanistically related, as both involve the production of siRNA [5].

RNAi can be used to engineer resistance in plants against viruses. Plants expressing a copy of a viral gene in sense and/or antisense orientation can show resistance upon infection with the virus (or other virus containing identical sequences) through RNAi. One of the first studies investigating DNA-directed RNAi in plants compared the ability of constructs expressing transcripts of sense, antisense and both polarities to yield resistance to an RNA virus in tobacco and silence an endogenous GUS reporter gene in rice [6]. In both cases it was shown that duplex RNA (expression of both polarities simultaneously) was more effective than expression of either sense or antisense RNA alone. Further studies showed that RNAi can be more efficiently induced using transgenes that express self-complementary "hairpin" (hp)RNA [7]. The hpRNA transgene is simply composed of a plant promoter and terminator between which an inverted repeat sequence of the target gene (sense and anti-sense) is inserted with a spacer region or intron between the repeats. The RNA transcribed from such a transgene hybridizes with itself to form a hairpin structure comprising a single-stranded loop region, encoded by the spacer region/intron, and a base-paired stem encoded by the inverted repeats, which mimics the dsRNA structure that induces RNAi. The whole length of the stem acts as a substrate for the generation of siRNAs, whereas the spacer region/intron is not involved in siRNA production but is required for the stability of the construct and appears to enhance the efficacy of silencing when directed against viruses [8].

Geminiviruses are single-stranded (ss)DNA viruses that affect a wide range of economically important crops throughout the warmer regions of the world [9-11]. Viruses of the family *Geminiviridae *are divided between the four genera (*Mastrevirus*, *Curtovirus*, *Topocuvirus *and *Begomovirus*) based on host range, genome organization and insect vector [12]. Although they are ssDNA viruses that replicate in the nucleus and have no dsRNA phase within their replication cycle, geminiviruses are known to trigger PTGS in plants with the production of virus-specific siRNA [13,14]. In contrast to RNA viruses, which can only be affected by PTGS, geminiviruses may be targeted by both PTGS and TGS. TGS is implicated when siRNAs corresponding to the promoter regions are produced that lead to methylation of the promoter and thus inhibition of transcription [15]. TGS was shown to be effective against the begomovirus *Mungbean yellow mosaic virus *(MYMV) in a transient assay [16].

The genus *Mastrevirus *encompasses viruses with monopartite genomes that are transmitted by leafhoppers. Although the majority of mastreviruses infect monocotyledonous plants, a small number infect dicots [17-21]. A group of closely related mastreviruses, that includes *Chickpea chlorotic dwarf Pakistan virus *(CpCDPKV), cause chickpea stunt disease (CSD) and also infect a number of other legume and non-leguminous crops. CpCDPKV causes significant losses to chickpea cultivation in Pakistan.

**Figure 1:**
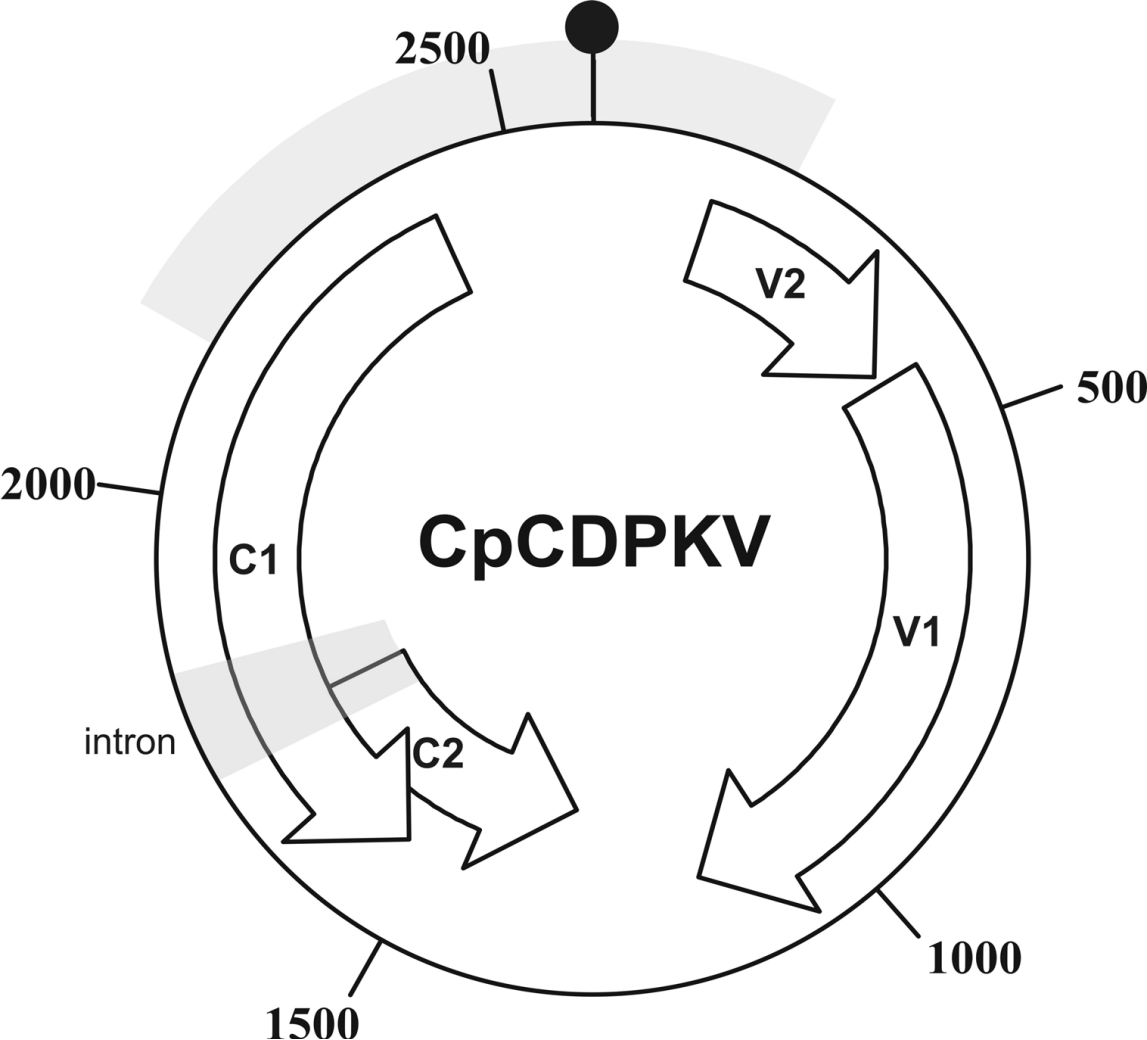
**Sequences of the CpCDPKV genome used to produce the hairpin-RNAi construct**. The diagram shows the circular DNA genome of CpCDPKV. Shown are the open-reading frames (ORFs) V1 and V2 (encoding the coat protein and movement protein, respectively), in the virion-sense and the C1 and C2 (encoding the replication associated protein [Rep; from a spliced product of the C1 and C2 ORFs] and the Rep A [from the C1 ORF], respectively), as well as the intron spliced from a mRNA from which Rep is translated. The large intergenic (non-coding) region (LIR) sits between the C1 and V1 ORFs, whereas the small intergenic region (SIR) sits between the C2 and V2 ORFs. The LIR contains a predicted hairpin-loop structure which contains (within the sequence of the loop) the conserved (between geminiviruses) TAATATTAC sequence which forms part of the origin of virion-strand DNA replication. The position of the hairpin-loop structure is indicated by the black circle at position zero. The CpCDPK virus sequences (730 bp) used to produce the hairpin RNAi construct is shown by the grey arc; this spans the 5' Rep, LIR and 5' MP sequences.

The genome of CpCDPKV, in common with all mastreviruses, contains four open reading frames (ORFs; Figure 1) [22]. Two ORFs (V1 and V2, encoding the coat protein [CP] and movement protein [MP], respectively) are encoded on the virion-sense strand and two ORFs (C1 and C2) are encoded on the complementary-sense strand [19]. The replication associated protein (Rep; the only virus-encoded protein required for viral DNA replication) is translated from a spliced mRNA, which fuses the C1 and C2 ORFs following the deletion of an intron, whereas the C1 ORF encodes the RepA protein, which is translated from an unspliced mRNA. The ORFs on the virion- and complementary-sense strands are separated by a large intergenic region (LIR) and a small intergenic region (SIR). The LIR contains a predicted hairpin-loop structure with the conserved (between geminiviruses) nonanucleotide motif (TAATATTAC), which forms part of the origin of virion-strand DNA replication, in the loop. The aim of the study described here was to investigate RNAi as a means of engineering resistance against CpCDPKV.

## Results

### Transgenic N. benthamiana plants are resistant to CpCDPKV infection

**Figure 2:**
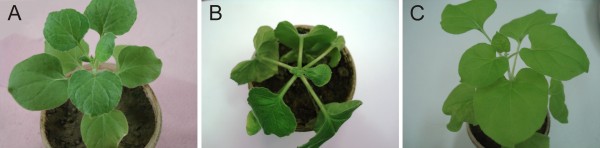
**RNAi-mediated resistance to CpCDPKV in transgenic *N. benthamiana***. Shown are a healthy non-inoculated *N. benthamiana *plant (A) and CpCDPKV-inoculated transgenic (B) and non-transgenic (C) *N. benthamiana *plants. Photographs were taken at 17 days post-inoculation.

A hpRNAi construct, containing sequences spanning the N-terminus of the Rep gene, the LIR and the N-terminus of the MP gene of CpCDPKV (Figure 1), was produced. *Nicotiana benthamiana *was transformed with the hpRNA construct (Chp6pFGC5941) by conventional *Agrobacterium*-mediated transformation and a single line, shown to harbor the construct by PCR amplification with the primers used to amplify the CpCDPKV fragments during construction, was selected for analysis. Both transgenic (25 plants) and non-transgenic (10 plants) *N. benthamiana *at the four true leaf stage were infiltrated with an *Agrobacterium *culture harboring a construct for the infectivity of CpCDPKV. All 10 non-transgenic plants showed symptoms of virus infection in the upper, newly emerging leaves at 14 days post-inoculation (dpi) consisting of foliar yellowing and curling (Figure 2B). Plants ceased to grow and died at approximately 25dpi. In contrast, all transgenic plants remained symptomless (Figure 2C), continued to grow and flowered, producing via seed (results not shown). These results are consistent with the hpRNA construct providing resistance to virus infection.

**Figure 3:**
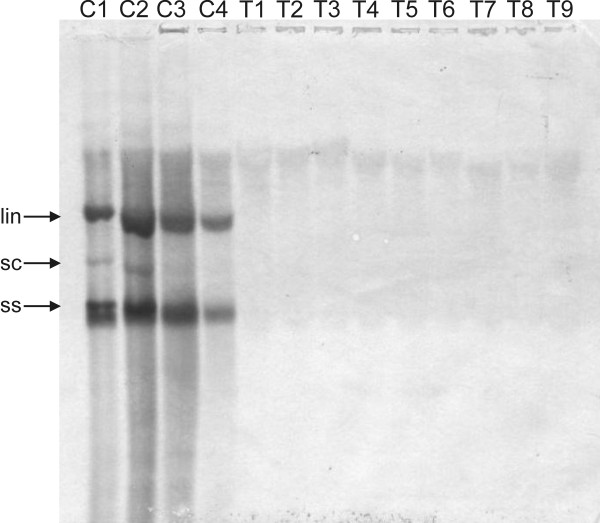
**Southern blot detection of CpCDPKV DNA in *N. benthamiana *plants**. The samples run on the gel were total DNA extracts (500 ng) from symptomatic, CpCDPKV infected non-transgenic *N. benthamiana *plants (lanes C1-C4) and transgenic *N. benthamiana *plants, harboring the hairpin RNAi construct, inoculated with CpCDPKV (lanes T1-T9). Viral DNA forms are indicated as supercoiled (sc), single-stranded (ss) and linear (lin).

Southern blot analysis of DNA samples extracted from the upper (non-inoculated) leaves of inoculated plants, probed for the presence of CpCDPKV DNA, showed the presence of high levels of typical ss and dsDNA viral forms in symptomatic, non-transgenic *N. benthamiana *plants (Figure 3). No hybridizing bands were detected in inoculated transgenic plants. These results suggest that transgenic expression of the hpRNAi construct was able to prevent symptomatic infection of plants by CpCDPKV.

### Determination of virus titer in inoculated plants by quantitative PCR

**Figure 4:**
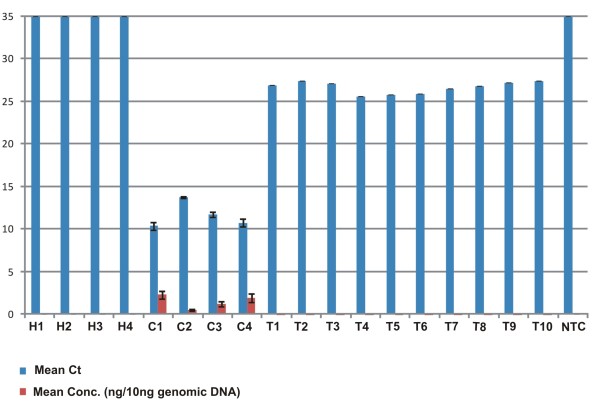
**Quantitative real-time polymerase chain reaction analysis of viral DNA levels in transgenic and non-transgenic, CpCDPKV -inoculated *N. benthamiana *plants**. Shown is a bar graph of threshold cycle (Ct) values (blue bars) and derived viral DNA concentrations (ng/10 ng genomic DNA; brown bars) for reactions with DNA extracted from healthy non-inoculated (H1-H4), infected non-transgenic (C1-C4) and inoculated transgenic (T1-T10) *N. benthamiana *plants. The results of a PCR reaction with no input (template) DNA is shown for comparison (NTC). Each bar is the mean of three repeated PCR reactions and the error bars indicate standard deviation.

The titre of CpCDPKV in inoculated transgenic and infected non-transgenic plants was determined by quantitative real-time PCR, the results of which are summarized in Figure 4 and Table 1. A standard curve was established using dilutions of the CpCDPKV clone and this was used to calculate viral DNA concentrations in plants by comparison of the threshold cycles (Ct) to the standard curve. The overall efficiency of these reactions was 98%.

**Table 1:** Quantitative real-time PCR estimation of viral DNA levels for CpCDPKV-inoculated transgenic and non-transgenic plants

Sample*	Mean Ct^@^	Mean viral DNA concentration^@ ^(ng/10 ng genomic DNA)	Standard deviation of CT	Standard deviation viral DNA concentration
Standard 1	6.92	10	2.087	0.0000
Standard 2	12.61	1	0.107	0.0000
Standard 3	17.33	0.1	0.257	0.0000
Standard 4	21.1	0.01	0.877	0.0000
Standard 5	26.07	0.001	1.207	0.0000
Healthy 1	35	0.0000	0.0000	0.0000
Healthy 2	35	0.0000	0.0000	0.0000
Healthy 3	35	0.0000	0.0000	0.0000
Healthy 4	35	0.0000	0.0000	0.0000
Control 1	10.36	2.3	0.678	0.4632
Control 2	13.78	0.46	0.899	0.0927
Control 3	11.7	1.22	1.008	0.295
Control 4	10.74	1.92	0.256	0.467
Transgenic 1	26.85	0.0000938	0.7862	0.0000303
Transgenic 2	27.36	0.0000825	0.5655	0.00003065
Transgenic 3	27.07	0.0000842	0.5603	0.00005189
Transgenic 4	25.56	0.000172	1.453	0.00007732
Transgenic 5	25.73	0.000159	0.8234	0.00002111
Transgenic 6	25.83	0.000152	0.3278	0.00003265
Transgenic 7	26.5	0.000111	0.9562	0.00002111
Transgenic 8	26.77	0.0000972	0.4445	0.00002241
Transgenic 9	27.16	0.0000814	0.8129	0.00002231
Transgenic 10	27.35	0.0000813	1.421	0.00001317
NTC	35	0.0000	0.0000	0.0000

*The samples are indicated as non-inoculated healthy *N. benthamiana *plants (Healthy 1-4), infected non-transgenic plants (Control 1-4) and inoculated transgenic plants (Transgenic 1-10). The results of PCR reactions with known amounts of viral input (template) DNA, used to produce the standard curve, are indicated (Standard 1-5). A reaction with no input (template) DNA (NTC) was included as a control.

**^@ ^**For each sample PCR reactions were conducted in triplicate and the mean value and standard deviations between the values, for both the threshold cycle (Ct) and mean concentrations (in ng viral DNA per 10 ng of plant genomic DNA) are given.

PCR reactions with DNA extracted from healthy, non-inoculated *N. benthamiana *plants and from a reaction with no input (template) DNA had Ct values equivalent to the total number of cycles used in the experiment, indicating that the threshold level was not achieved and thus that these reactions contained no detectable viral DNA. In contrast, four CpCDPKV-infected control (non-transgenic) *N. benthamiana *plants were shown to contain relatively large amounts of viral DNA (between 0.46 and 2.3 ng per 10 μg of genomic DNA). This relatively large variation (~5 fold) in viral DNA levels between different infected plants likely results from the plants being at different infection stages at the time of sampling (meaning that differing numbers of cells were infected).

Although Southern blot analysis was unable to detect viral DNA in the inoculated transgenic plants, the quantitative PCR analysis showed the presence of viral DNA at between 0.0813 and 0.172 pg per 10 μg of genomic DNA. This is only an ~2 fold variation, meaning that the transgenic plants showed less variability in viral DNA levels, even though more plants were examined. However, the difference in viral DNA levels between transgenic and non-transgenic plants was striking - between 2,600 and 28,000 fold.

To assess the specificity of the PCR, a melt curve analysis of the resulting PCR products was conducted (Additional file 1). The analysis showed only a single peak, indicative of the melting of the PCR product at a single temperature, showing that only a single product was amplified.

## Discussion

The RNAi approach has been investigated extensive as a means of delivering resistance to begomoviruses in plants [23,24]. In at least one case this has led to the first successful field tests of RNAi-based resistant plant lines [25,26]. However, the lack of success of other studies has led to some ask whether this approach can, ultimately, succeed; with virus diversity and virus-encoded suppressors of RNAi as possible negative indicators of the ultimate success of the RNAi-based approach [27,28]. For mastreviruses, specifically the monocot-infecting *Maize streak virus*, a protein-mediated resistance approach has proven successful [29] and recently, using a transient cell culture assay, the potential usefulness of the RNAi approach to MSV resistance was shown [30]. Here we have shown that stable integration of a hpRNAi construct provides *N. benthamiana *with resistance to a dicot-infecting mastrevirus.

Although all transgenic plants remained symptomless and Southern blot analysis did not show the presence of viral DNA, quantitative PCR analysis was able to show the presence of very low viral DNA titers; a 2,600 to 28,000 fold difference between transgenic and non-transgenic plants. These results are consistent since PCR is a far more sensitive technique than Southern blotting, indicating that virus DNA levels in transgenic plants are below the detection threshold of Southern blotting. This finding is very similar to the results obtained for infections of the DNA A components of many bipartite begomoviruses in the absence of the DNA B. For bipartite begomoviruses both the DNA A and DNA B components are required for symptomatic infections of plants [31,32]. Nevertheless, for most bipartite begomoviruses examined, the DNA A component can spread systemically in plants in the absence of the DNA B [33-35]. Plants infected with only DNA A exhibit no symptoms and reduced viral DNA levels. These results suggested that, following *Agrobacterium*-mediated inoculation, the DNA A is able to gain access to the phloem and spread throughout the plant (either as virions or a nucleoprotein complex involving the CP), but is unable to reestablish infection in the upper (younger, actively growing) parts of the plant in the absence of the genes products encoded by DNA B (which are involved in virus intra- and intercellular movement [36]. The RNAi construct used here, by virtue of containing sequences of both the MP gene and its promoter, should induce both PTGS and TGS of the viral MP gene. It is thus possible, in a manner analogous to the situation with DNA A only infections, that RNAi-mediated down-regulation of MP expression prevented efficient spread of the virus from the site of inoculation to young, actively growing tissues of the plant.

An alternative, and possibly more plausible, explanation of the phenomenon seen in the hpRNAi construct containing plants is that virus replication (and possibly also movement) is severely impaired. However, possibly minute amounts of virus can move into the phloem and spread throughout the plant, but at levels that are insufficient to reestablish infection of cells in the upper (younger, actively growing) parts of the plant - cells which also harbor the hpRNAi construct. The lack of variability in DNA levels in the younger tissues of inoculated transgenic plants (~2 fold in comparison to ~5 fold in non-transgenic infected plants) might suggest that viral DNA replication is not occurring, or occurring at very low levels, in these tissues.

Further studies will address the precise mechanism of the resistance by, for example looking for small RNAs derived from the transgene and whether TGS, in addition to PTGS, is involved. Additionally the ability of the hpRNAi construct, derived from CpCDPKV, will be assessed for its ability to provide protection to other related viruses; such as *Chickpea chlorotic dwarf Sudan virus *and the recently identified *Chickpea chlorotic dwarf Syria virus *[21]. Ultimately the aim is to produce chickpea lines harboring an hpRNAi construct which can provide a broad spectrum resistance to these legume-infecting mastreviruses.

## Methods

### Production of a hairpin RNAi construct against CpCDPKV

For CpCDPKV one hpRNA construct, Chp6pFGC5941, was designed. This construct was based on a part of Rep (that is also a part of RepA) gene, part of the movement protein (MP) gene and the LIR (Figure 1). Specific sets of primers (Table 2) were designed with suitable restriction sites for the PCR amplification of a DNA fragment of about 730 bp in sense and antisense orientations. A clone of CpCDPKV (acc. no. AM849097 [19]) was used as template in PCR. These PCR products were separately cloned into the T/A cloning vector pTZ57R/T to yield pChP6S and pChP6AS. For cloning the gene fragment in the RNAi vector pFGC5941 in sense orientation, the fragment was excised from pChP6S by using *Xho*I and *Nco*I and ligated in suitably restricted pFGC5941 to produce pChP6SRNAi. Then a full fragment was excised from the pChP6AS in antisense orientation by using *Xba*I and cloned in pChP6SRNAi to yield the final hpRNAi construct ChP6pFGC5941. The integrity of the construct was confirmed by restriction digestion and sequencing (results not shown).

**Table 2:** Oligonucleotide primers used in the production of the RNAi construct

Primer	Orientation	Primer sequences^#^	Cloning sites*
Chp6SF	sense/forward	5'CCAACTCGAGTTATCAAGCTGGACAAGAGC 3'	*Xho*I
Chp6SR	sense/reverse	5'CCTCCCATGGTTCGCCTTAAACAGAAGG 3'	*Nco*I
Chp6ASF	antisense/forward	5'CCTCTCTAGATTCGCCTTAAACAGAAGG 3'	*Xba*I
Chp6ASR	antisense/reverse	5'CCAATCTAGATTATCAAGCTGGACAAGAGC 3'	*Xba*I

* Restriction endonuclease recognition sequences introduced into the primers to facilitate cloning of fragments into pFGC5941.

^# ^The introduced restriction endonuclease recognition sequences are underlined.

### Plant transformation

*N. benthamiana *was transformed with the RNAi construct (ChP6pFGC5941) by the leaf disc method using *Agrobacterium tumefaciens *strain LBA4404 [37,38]. Integration of the construct in the transgenic line was confirmed by PCR using the primer sets used to produce the construct (Table 2). The transgene showed no adverse effect on the growth of plants with all transgenic plants showing a normal phenotype and producing viable seed (results not shown).

### Inoculation of plants with CpCDPKV

Twenty-five T_1 _transgenic *N. benthamiana *plants harboring the RNAi construct, obtained from the self-pollination of T_o _plants, were agro-infiltrated with a construct for the infectivity of CpCDPKV as described previously [19]. Ten non-transgenic plants of the same age (4 weeks old) were infiltrated as controls. All agro-infiltrated plants were observed periodically for the appearance of symptoms. The presence of virus in transgenic and non-transgenic plants was detected by Southern hybridization and real-time PCR.

### Southern hybridization for the detection of viral DNA

Total DNA was isolated from the inoculated plants [39] and 500 ng of DNA was resolved on 1% agarose gel in 0.5 × TAE buffer. Blotting was performed as described earlier [40]. The blot was hybridized with a 1000 bp biotin-labeled DNA probe (prepared using a Biotin DecaLabel™DNA labeling kit [Fermentas]) containing portions of genes encoding movement protein (MP) and coat protein (CP). The hybridization and DNA detection was performed according to the manufacturer's recommendations.

### Real-time PCR quantification of viral DNA in plants

Quantitative PCR (qPCR) was performed for the quantification of viral DNA in transgenic plants (showing resistance against CpCDPKV) and non-transgenic plants (showing infection for CpCDPKV) by using SYBER green dye (IQ SYBER Green supermix, BIO-RAD USA) as described previously [41]. Based on the nucleotide sequence of CpCDPKV (AM849097), a primer pair QF (5'TAAAAGGCGCACTAATGGGTAGACCGTAGA3' - spanning nucleotide coordinates 102-131) and QR (5'GGCGATAACCACCTTCCCG3' - spanning nucleotide coordinates 251-233) was designed to amplify a product of 150 bp specific to CpCDPKV. Young leaf samples were collected from the inoculated plants at 17 dpi and qPCR was performed in three replications along with the standards and controls.

## Competing interests

The authors declare that they have no competing interests.

## Authors' contributions

NN, IA performed the experiments. RWB and SM conceived the study. NN, IA, RWB and SM wrote the manuscript. All authors read and approved the final manuscript.

## Supplementary Material

Additional file 1 **Melt curve analysis of the products produced during the qPCR analysis**. The graph shows a plot of the negative derivative of fluorescence versus temperature (°C) for each amplification tube. A single peak is evident, indicative of the amplification of a single product. Click here for file
