# Supplementary figures and images for: RNA interference-based resistance against a legume mastrevirus
Source: Virol J. 2011 Nov 2;8:499. doi: 10.1186/1743-422X-8-499 (PMC3214926; doi:10.1186/1743-422X-8-499)

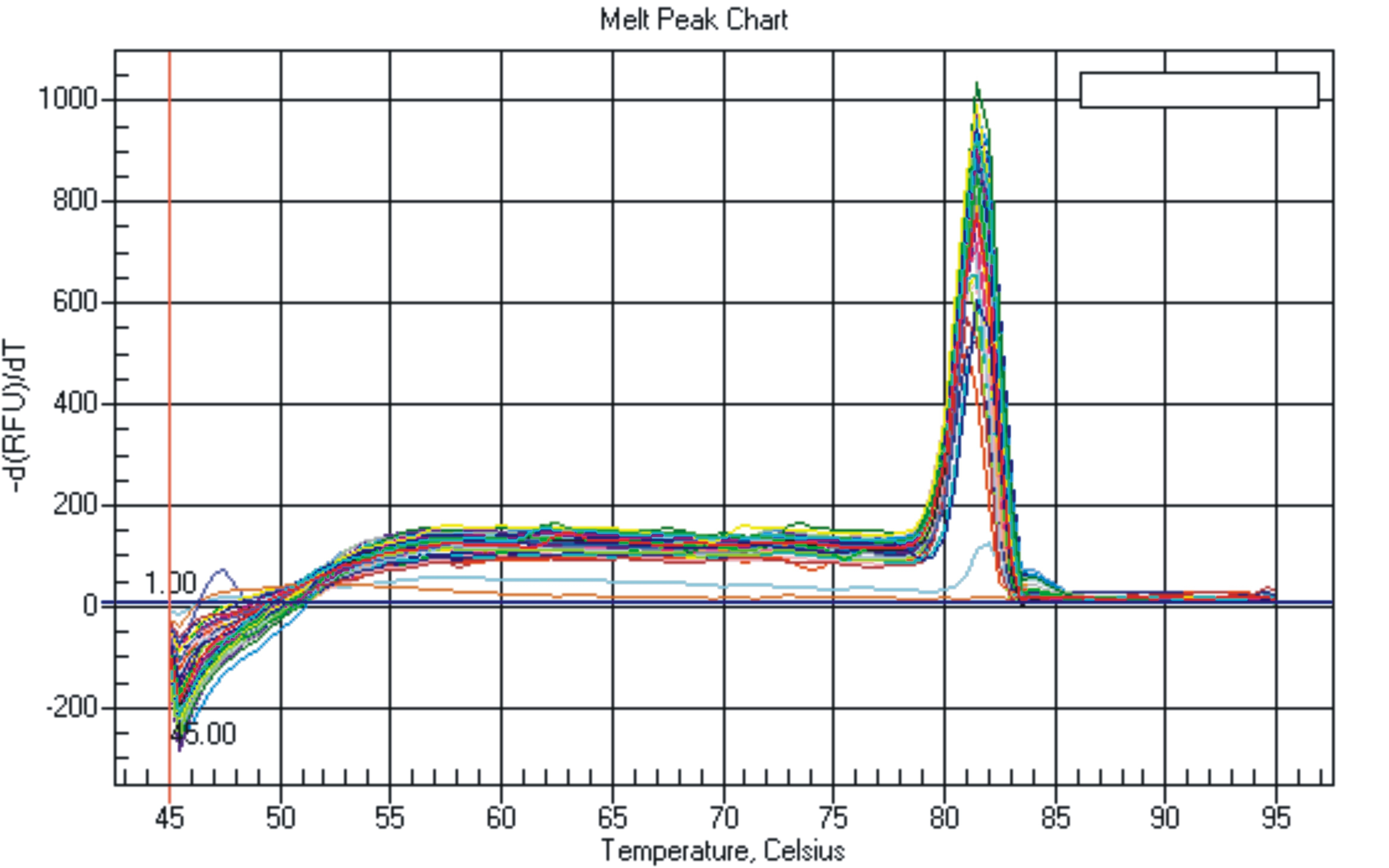

Supplement: Additional file 1 — Melt curve analysis of the products produced during the qPCR analysis. The graph shows a plot of the negative derivative of fluorescence versus temperature (°C) for each amplification tube. A single peak is evident, indicative of the amplification of a single product. [file 1743-422X-8-499-S1.JPEG]
